# A novel mutation of the RPGRIP1L gene in a Chinese boy with Joubert syndrome with oculorenal involvement

**DOI:** 10.1186/s12887-023-04415-1

**Published:** 2023-11-23

**Authors:** Qian Li, Qianying Liu, Suwen Liu, Lichun Yu, Zhenle Yang, Cong Wang, Jing Wang, Shuzhen Sun

**Affiliations:** 1 Department of Pediatric Nephrology and Rheumatism and Immunology, Shandong Provincial Hospital, Cheeloo College of Medicine, Shandong University, No. 324 Jingwu Road, Huaiyin District, Jinan, 250021 People’s Republic of China; 2 Department of Pediatric Nephrology and Rheumatism and Immunology, Shandong Provincial Hospital Affiliated to Shandong First Medical University, No. 324 Jingwu Road, Huaiyin District, Jinan, 250021 People’s Republic of China

**Keywords:** Joubert syndrome, Child, Developmental delay, Ataxia, End-stage kidney disease, Strabismus

## Abstract

**Background:**

Joubert syndrome (JS) is a rare genetically heterogeneous primary ciliopathy characterized by a pathognomonic cerebellar and brainstem malformation, the “molar tooth sign”, and variable organ involvement (such as eye, kidney, liver, and skeleton). Here, we present a case of JS in a Chinese boy.

**Case presentation:**

An 11-year-old Chinese boy presented with neonatal asphyxiation and hypoxia, strabismus, subsequent developmental delay, ataxia and end-stage kidney disease (ESKD). Routine blood tests showed severe anemia, increasing blood urea nitrogen and creatinine, elevated parathyroid hormone, hypocalcemia, hypokalemia and metabolic acidosis. Urine tests showed mild proteinuria. Ultrasound showed two small kidneys. Brain magnetic resonance imaging (MRI) showed dysplasia of the cerebellar vermis and extension of the upper cerebellar feet with the “molar tooth sign”. Genetic analysis showed novel compound heterozygous mutations in the RPGRIP1L gene [p.L447fs*7(p.Leu447fsTer7) and p.G908V (p.Gly908Val)].

**Conclusion:**

In the present study, we identified novel compound heterozygous mutations in the RPGRIP1L gene in a Chinese boy. The clinical and genetic findings of this study will expand the understanding of JS.

## Background

Joubert syndrome (JS; OMIM PS213300) is a rare congenital neurodevelopmental primary ciliopathy. Its population-based prevalence reaches 1.7 per 100,000 in patients ranging from 0 ~ 19 years of age [1]. It is typically characterized by a pathognomonic malformation of the midbrain–hindbrain junction called the “molar tooth sign” (MTS). The common manifestations include hypotonia, abnormal eye movements, developmental delay and episodic breathing dysregulation during early childhood, cerebellar ataxia and cognitive impairment [2]. Two-thirds of individuals with JS present with variable involvement of other organs, including retinal dystrophy, renal disease, ocular colobomas, occipital encephalocele, hepatic fibrosis, polydactyly, oral hamartomas, and endocrine abnormalities at different ages and with variable severity [3].

Here, we present a case of JS in a Chinese boy with a novel compound heterozygous RPGRIP1L (retinitis pigmentosa GTPase regulator interacting protein 1-like gene, NM_015272) gene mutation.

## Case presentation

An 11-year-old Chinese boy was referred to our hospital with complaints of weight loss greater than 5 kg over six months, accompanied by pallor, fatigue, poor appetite and lower limb twitching at night. He was the first child of healthy parents and was delivered vaginally at full term, with a birth weight of 3.7 kg. Asphyxiation and hypoxia occurred at birth. His developmental milestones were delayed, and he was diagnosed with cerebral palsy, hydrocephalus and strabismus of the left eye. Surgery was performed to resolve tongue tie and slurred speech at age 3. The patient had undergone language rehabilitation treatment for 3 years. There was no family history. Physical examination revealed normotension, cachexia with height 154 cm and weight 25 kg, slow reaction, dysarthria and unsteady gait without nystagmus or hand tremor.

**Fig. 1:**
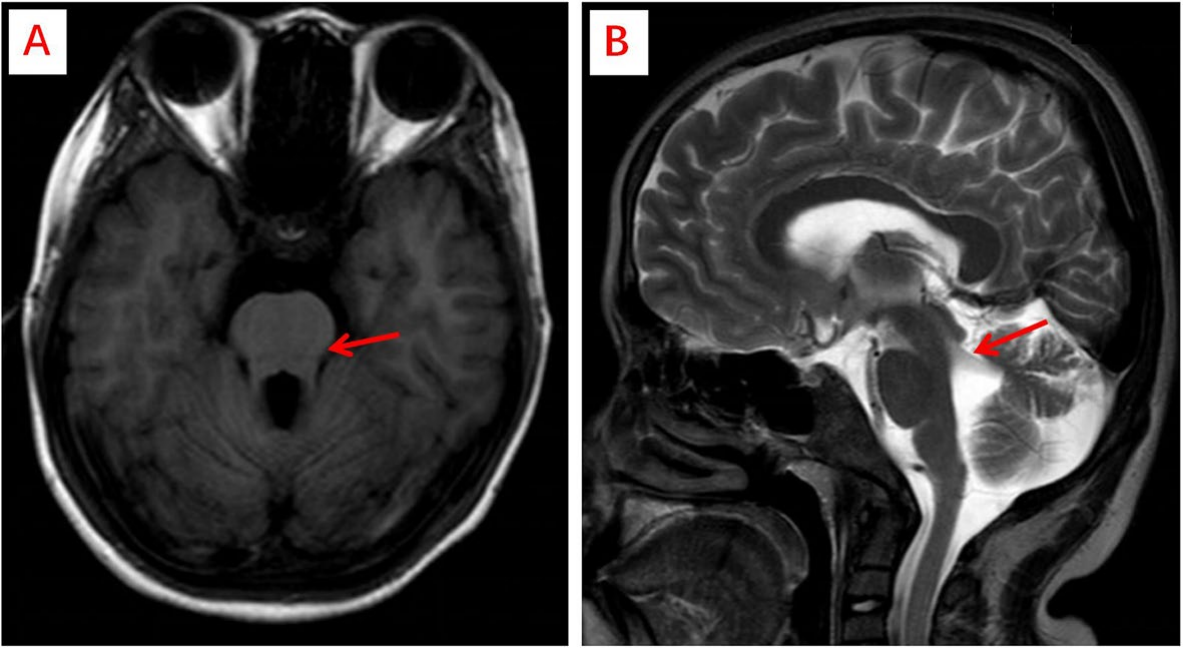
Brain MRI showed classic findings of Joubert syndrome. **A** shows extension of the upper cerebellar feet with the “molar tooth sign” (arrow); (**B**) shows a thickened upper cerebellar foot almost perpendicular to the brainstem (arrow)

The boy underwent detailed examinations after admittance to our department. Routine blood tests showed severe anemia (hemoglobin 56 g/L). Urine tests showed mild proteinuria; the 24-h urinary protein quantification was 0.61 g (24.4 mg/kg). Renal function tests showed increased blood urea nitrogen (67.8 mmol/L) and creatinine (746.70 μmol/L), and the estimated glomerular filtration rate (eGFR) was 7.53 ml/(min•1.73 m^2^). Blood tests showed significantly elevated parathyroid hormone (PTH) (1073.00 pg/ml), hypocalcemia (0.49 mmol/L), hypokalemia (2.99 mmol/L), and metabolic acidosis. Antinuclear antibody (ANA) was positive (1:320), while other autoantibodies were negative. The level of complement 3 was low (0.63 g/L), and the level of complement 4 was normal. The results of liver function, thyroid function, carcinoembryonic antigen, alpha-fetoprotein, hemoglobin A1c, blood glucose, thyroid ultrasound, abdominal ultrasound and cardiac ultrasound and chest CT were normal. Ophthalmic examination indicated myopia in both eyes and a leopard pattern under the fundus. Hearing was normal in both ears. Renal ultrasound showed two small kidneys. Brain magnetic resonance imaging (MRI) showed dysplasia of the cerebellar vermis and extension of the upper cerebellar feet with an MTS (Fig. 1).

**Fig. 2:**
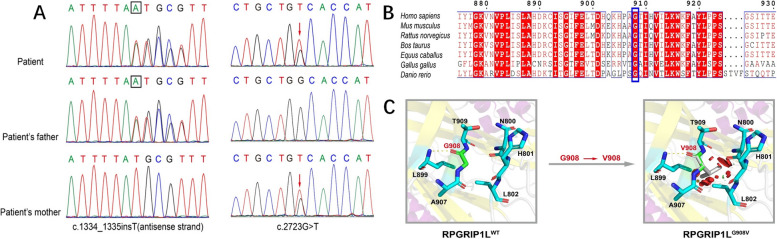
Identification and characterization of RPGRIP1L variants. **A** Sanger sequencing confirmed the compound heterozygous mutation of the RPGRIP1L gene of the proband inherited from the father and mother. **B** Conservation prediction plots of c.2723G > T (p.G908V). **C** Predicted three-dimensional models of wild-type and mutant (p.G908V) RPGRIP1L

DNA was obtained from peripheral blood from the patient and his parents and submitted for trio whole-exome sequencing (trioWES) to Chigene Co., Ltd. Genetic analysis showed novel compound heterozygous mutations in the RPGRIP1L gene [c.1334_c.1335insT (p.L447fs*7) and c.2723G > T (p.G908V)]. His parents were heterozygous carriers and did not present any symptoms. Gene sequencing revealed that the father carried the c.1334_c.1335insT variant, and the mother carried the c.2723G > T variant. According to the American College of Medical Genetics and Genomics (ACMG) criteria, the c.1334_c.1335insT variant of the RPGRIP1L gene was classified as a likely pathogenic variant (PVS1 + PM2) because this loss-of-function (LOF) variant could lead to a possible loss of gene function, and the c.2723G > T variant was classified as a variant of uncertain significance (PM2 + PM3 + PP3). Both conservation prediction plots and predicted three-dimensional models predicted that the substitution of amino acids (p.G908V) in the mutated protein would lead to a change in function and eventually result in disease (Fig. 2).

The boy was given supportive treatment, including polysaccharide iron complex, calcium, calcium carbonate D3, sodium bicarbonate and human erythropoietin injection. Kidney transplantation was performed 3 months later.

## Discussion

We reported a Chinese boy with JS who presented with neonatal asphyxiation and hypoxia, cerebral palsy, strabismus, subsequent developmental delay, ataxia and end-stage kidney stage (ESKD). Brain imaging showed dysplasia of the cerebellar vermis and extension of the upper cerebellar feet with an MTS. This case was caused by a novel compound heterozygous mutation of the RPGRIP1L gene.

The diagnosis of JS is based on three primary criteria: (1) the MTS on MRI, (2) hypotonia in infancy and (3) developmental delay or intellectual disability [4]. The most common involvement of JS is neurological involvement,such as development lag, intellectual disability, dystonia, abnormal eye movements, ataxia, abnormal respiratory rhythm, and the second is renal involvement, such as ESKD, hematuria, albuminuria, diffuse renal disease, renal cystic lesions, enhanced echogenicity in parenchyma. Therefore combining ultrasound with MRI, urine test could improve an earlier diagnosis of JS. The patient was diagnosed as Joubert syndrome (JS) definitely according to the clinical manifestations and related examinations. Genetic analysis showed novel compound heterozygous mutations in the RPGRIP1L gene. The two new sites was regarded as a likely pathogenic variant and a variant of uncertain significance respectively, according to ACMG criteria. RPGRIP1L has been reported to be closely related to renal damage of JS in the past.Therefore, the boy was diagnosed with JS with an RPGRIP1L mutation. However, it was a pity that functional testing was not done to verify the compound heterozygous mutation.

JS is classified into eight different phenotypic subgroups: pure JS, JS with ocular involvement, JS with renal involvement, JS with oculorenal involvement, JS with hepatic involvement, JS with orofaciodigital involvement, JS with acrocallosal features and JS with Jeune asphyxiating thoracic dystrophy, according to the presence of associated extra-central nervous system (CNS) features [5]. Our patient presented with strabismus and ESKD, so he was regarded as JS with oculorenal involvement.

JS is characterized by extreme genetic heterogeneity, with more than 40 causative genes, all of which encode proteins responsible for the formation or functioning of the primary cilium [6]. JS is mainly inherited in an autosomal recessive fashion, although there is an X-linked recessive form caused by pathogenic variants in the OFD1 gene [7] and an autosomal dominant form caused by truncating or splice-site variants in the SUFU gene [8]. CEP290, NPHP1, AHI1, OFD1, RPGRIP1L, CC2D2A, TMEM67, TMEM216, TMEM138, and TMEM237 have been reported to be associated with kidney disease. The RPGRIP1L gene, also known as the NPHP8 gene, which is one of the genes that causes nephronophthisis (NPHP, MIM 256100), deserves special mention. It is located at 16q12.2 and encodes a protein (RPGRIP1L) that localizes to the basal bodies and transition zone of primary cilia. It is essential for the function of primary cilia and plays an important role in fundamental embryonic development, including the development of the retina, nervous system, kidneys, bile ducts, and limbs [9]. RPGRIP1L is required for normal brain development and kidney function. Previous reports have shown that RPGRIP1L gene mutation is associated with cerebellar vermis hypoplasia, intellectual disability/development delay, NPHP, and ocular motor apraxia (OMA) in JS patients [10]. A clear genotype–phenotype correlation seems to exist in the case of RPGRIP1L, with only one truncating mutation or a homozygous missense mutation causing NPHP, whereas homozygous truncating mutations cause JS-related disorders [11].

NPHP constitutes the most frequent genetic cause of ESKD in children and young adults. Up to 25–30% of JS individuals develop renal disease, mainly presenting as juvenile NPHP, which may remain asymptomatic for several years [12]. Wolf MT et al. studied a cohort of 56 patients from central Europe and the United States with NPHP and identified four different mutations in the RPGRIP1L gene in five different families [13]. Takagi Y reported 11 patients with JS and ESKD who underwent renal replacement therapy and found that one patient carried mutations in the RPGRIP1L gene [14]. Ying L reported 17 Chinese children with JS and discovered that 3 of 6 cases complicated with ESKD had compound heterozygous mutations in the RPGRIP1L gene [15].

In this study, the patient was misdiagnosed until he presented with weight loss, severe anemia, mild proteinuria, elevated parathyroid hormone (PTH), hypocalcemia, hypokalemia, and metabolic acidosis secondary to ESKD at the age of 11. The reasons for misdiagnosis mainly included family neglect and unawareness of JS. The disease is a rare genetic developmental disorder. An estimated incidence of JS is 1/100,000 to 1/8000. Many people, even many doctors do not know or even hear of the disease. However, with the rapid development of medical technology and gene detection methods in recent years, the detection rate of JS is increasing year by year. We should pay more attention to neurological and kidney involvement, and gene detection should be suggested for those suspected patients. This kind of genotype–phenotype correlation may help to guide individualized management in JS patients.

## Conclusion

The present study identified a novel compound heterozygous mutation, p.L447fs*7 (p.Leu447fsTer7) and p.G908V (p.Gly908Val), in the RPGRIP1L gene in a Chinese boy. The clinical and genetic findings of this study will expand the understanding of JS.

## Data Availability

The original contributions presented in the study are included in the article, and further inquiries can be directed to the corresponding author on reasonable request.
